# Serum Exosomal Circular RNA Expression Profile and Regulative Role in Proliferative Diabetic Retinopathy

**DOI:** 10.3389/fgene.2021.719312

**Published:** 2021-08-10

**Authors:** Xinsheng Li, Jingfan Wang, Huiming Qian, Yan Wu, Zhengyu Zhang, Zizhong Hu, Ping Xie

**Affiliations:** Department of Ophthalmology, The First Affiliated Hospital of Nanjing Medical University, Nanjing, China

**Keywords:** proliferative diabetic retinopathy, exosome, circular RNA, angiogenesis, bioinformatics analysis

## Abstract

**Background:**

Proliferative diabetic retinopathy (PDR), as one of the main microvascular complications of diabetes mellitus, seriously threatens the visual function of the working-age population; yet, the underlying pathogenesis is still poorly understood. This study aimed to identify the distinct exosomal circular RNA (circRNA) expression in PDR serum and preliminarily explore the potential pro-angiogenic mechanism of specific exosomal circRNAs.

**Methods:**

We collected serum samples from 10 patients with PDR and 10 patients with age-matched senile cataract to detect the exosomal differentially expressed genes (DEGs) of circRNAs *via* high-throughput sequencing, followed by validation with quantitative real-time PCR (qRT-PCR). Next, bioinformatics analyses including competitive endogenous RNA (ceRNA) network, protein–protein interaction network (PPI), and functional enrichment analyses were performed. In addition, the potential function of circFndc3b (hsa_circ_0006156) derived from high-glucose-induced endothelial cells was analyzed in human retinal vascular endothelial cells (HRVECs).

**Results:**

In total, 26 circRNAs, 106 microRNAs (miRNAs), and 2,264 messenger RNAs (mRNAs) were identified as differentially expressed in PDR serum exosomes compared with cataract serum exosomes (fold change > 1, *P* < 0.05). A circRNA–miRNA–mRNA ceRNA network was established. Kyoto Encyclopedia of Genes and Genomes (KEGG) pathway analysis revealed that the mRNAs were mainly enriched in the PI3K–Akt signaling pathway, MAPK signaling pathway, Wnt signaling pathway, and VEGF signaling pathway. The PPI network and module analysis identified 10 hub genes, including *RhoA*, *Cdc42*, and *RASA1*. Finally, circFndc3b and exosomes derived from high-glucose-induced endothelial cells were identified with the capability to facilitate angiogenesis *in vitro*.

**Conclusion:**

Aberrant profiling of exosomal circRNAs in PDR serum was identified. CircFndc3b derived from high-glucose-induced endothelial cells may play an important role in the angiogenesis of PDR.

## Introduction

Diabetes mellitus (DM), as the third common chronic metabolic disorder, has become a major public health challenge worldwide (Maffi and Secchi, 2017). Hyperglycemia can constantly damage capillaries in any part of the body. Diabetes-associated retinal complication is usually known as diabetic retinopathy (DR), which is a leading cause of visual impairment and blindness among the working-age population in developed countries (Leasher et al., 2016). According to the presence or absence of retinal neovascularization, DR is generally classified into non-proliferative diabetic retinopathy (NPDR) and proliferative diabetic retinopathy (PDR). For PDR, although therapeutic strategies, such as retinal photocoagulation, intraocular injection of anti-vascular endothelial growth factor (anti-VEGF), or even pars plana vitrectomy (PPV), have been applied in the clinic, the recovery of vision is still unsatisfactory (Wang and Lo, 2018).

Intercellular communication is a crucial component in all multicellular organisms to maintain homeostasis and synergistically resist potential pathological threats. Exosomes (nanosized membrane vesicles) secreted from almost all cell types widely take part in cell-to-cell communication. Donor cells can modulate the biological function of recipient cells by transmitting molecular cargo, such as proteins, lipids, and nucleic acids, *via* exosomes (Fanale et al., 2018). In 1976, the first circular RNA (circRNA) was isolated and identified from virus (Sanger et al., 1976). circRNA is a novel type of endogenous non-coding RNA (ncRNA) characterized by covalent closed-loop structures. CircRNAs can act as microRNA (miRNA) sponges and inhibit the negative regulation of miRNA on its target messenger RNAs (mRNAs) by competitively binding to miRNAs through the miRNA response element (MRE), which is called the competitive endogenous RNA (ceRNA) hypothesis (Chen and Yang, 2015). Besides, circRNAs are also capable of interacting with proteins and affect their functions (Tang et al., 2021). Recently, circRNAs were identified as important cargos loaded in exosomes. Li et al. (2015) performed RNA sequencing (RNA-seq) analyses of liver cancer cells and cell-derived exosomes, presenting abundant and stable expression patterns of circRNAs in exosomes. Until now, a number of exosomal circRNAs have been identified as meaningful biomarkers of disease (Li et al., 2020; Wu et al., 2020). Meanwhile, more studies have linked exosomes with circRNAs to widely investigate the roles of exosomal circRNAs in the development and progression of diseases (Chen et al., 2020; Yan et al., 2020). However, few research have reported on PDR serum exosomes.

The present study focused on the differential expressions of circRNAs in serum exosomes between the PDR group and the age-matched group. Bioinformatics analysis and *in vitro* study were used to explore the potential mechanisms that differential exosomal circRNAs might be involved in in PDR. We hope that this work can identify some meaningful biomarkers in order to provide an explicit reference for the further investigation of exosomal circRNAs in PDR.

## Materials and Methods

### Patient Selection and Sample Collection

We recruited 20 individuals, 10 DM patients with PDR and 10 age-matched senile cataract patients as the control group, from the First Affiliated Hospital of Nanjing Medical University from March 2020 to April 2020. The diagnosis of PDR was in accordance with the criteria of the American Ophthalmology Association (Lechner et al., 2017). Blood samples were collected at least 12 h after a meal, followed with standing for 2 h at 4°C, then centrifuging at 2,000 × *g* for 20 min at 4°C, discarding precipitation. At least 3 ml serum was collected from each sample and stored at –80°C.

### Identification of Exosomes

For transmission electron microscopy (TEM), 10 μl of resuspended exosomes was loaded in 200-mesh carbon-coated copper grids for 1 min, dried in air, negatively stained with 1% phosphotungstic acid for 20 s, blotted free of redundant liquid, and dried under a lamp. Subsequently, the grids were detected using an H7650 electron microscope (Hitachi, Tokyo, Japan). Nanoparticle tracking analysis (NTA) of isolated exosomes was measured using a ZETASIZER Nano series-Nano ZS (Malvern Panalytical, Malvern, United Kingdom) by Ribo (Guangzhou, China). Briefly, 1 ml phosphate-buffered saline (PBS) was added to the resuspended exosomes and the mixture slowly injected into a clean particle-free sample pool, avoiding the formation of bubbles. The sample pool was covered and placed into the instrument. Manipulations were performed in accordance with standard protocols. For flow cytometry (FCM) of exosomes, exosomal preparations were stained with FITC-labeled anti-CD63 and anti-CD81 monoclonal antibodies (BD Biosciences, Piscataway, NJ, United States) and analyzed using Accuri C6 Flow Cytometer (Becton Dickinson, Franklin Lakes, NJ, United States).

### Isolation of Exosomes, Extraction of Total RNAs, and High-Throughput Sequencing

Exosomes were isolated from serum using Invitrogen^TM^ Total Exosome Isolation Kits (Ribo, Guangzhou, China). The exosome isolation reagent was mixed well with the serum, then the mixture was centrifuged at 15,000 × *g*, 4°C, for 2 min after standing for 30 min, and the supernatant was discarded to obtain exosome precipitation. Total RNA was isolated from exosomes using the TRIzol reagent (Invitrogen, Carlsbad, CA, United States) according to the manufacturer’s instruction. RNA purity was assessed using ND-1000 NanoDrop, which required ratios of d*A*_260_/*A*_280_ ≥ 1.8 and *A*_260_/*A*_230_ ≥ 2.0. RNA integrity was evaluated using Agilent 2200 TapeStation (Agilent Technologies, Santa Clara, CA, United States), requiring RIN ≥ 7.0. Briefly, ribosomal RNAs (rRNAs) were removed from total RNA using an Epicenter Ribo-Zero rRNA Removal Kit (Illumina, San Diego, CA, United States), and then RNA was treated with RNase R (Epicenter, Madison, WI, United States) and fragmented to approximately 200 bp. Subsequently, the purified RNA fragments were subjected to first-strand and second-strand complementary DNA (cDNA) synthesis followed by adaptor ligation and enrichment with a low cycle according to the instructions in the NEBNext^®^ Ultra^TM^ RNA Library Prep Kit for Illumina (NEB, Ipswich, MA, United States). The purified library products were evaluated using the Agilent 2200 TapeStation and Qubit^®^2.0 (Life Technologies, Carlsbad, CA, United States) and then sequenced on HiSeq 3000 with 2 × 150 bp mode to identify differentially expressed genes (DEGs), including DEcircRNAs, DEmiRNAs, and DEmRNAs.

### CeRNA Network, PPI Network Construction, Identification of Hub Genes, and Functional Enrichment Analysis

We used the Cancer-Specific CircRNA Database^1^ to screen the target miRNAs of the DEcircRNAs. These target miRNAs were further filtered by the DEmiRNAs obtained from RNA-seq. Then, the target genes of these DEmiRNAs were predicted using the TargetScan, miRDB, and miRTarBase databases. Only the target genes predicted by these three databases were retained, which were then overlapped with the DEmRNAs collected from RNA-seq. Finally, according to the predicted relationship of circRNA–miRNA–mRNA, the ceRNA network was constructed using the Cytoscape software.^2^ The Search Tool for the Retrieval of Interacting Genes database^3^ was used to build the protein–protein interaction (PPI) networks of DEmRNAs. In this study, the criterion was set as an interaction score greater than 0.9. Subsequently, the hub genes were identified using the cytoHubba plug-in of the Cytoscape software (v3.7.0). In order to identify the possible function of the mRNAs in the ceRNA network, Gene Ontology (GO) and Kyoto Encyclopedia of Genes and Genomes (KEGG) pathway analyses were performed using the “clusterProfiler” package in R. A *P*-value < 0.05 was considered statistically significant.

### Validating the Expression of Dysregulated Exosomal circRNAs

Based on high-throughput RNA-seq, we further recruited six independent patients to verify the most upregulated expressions of exosomal circFndc3b (hsa_circ_0006156), circFAM13B (hsa_circ_0001535), and circRBM33 (hsa_circ_0001772). Since endothelial cells are considered to be the main cellular targets of diabetes-induced vascular damage (Hammes et al., 2011; Sheikh et al., 2014), quantitative real-time polymerase chain reaction (qRT-PCR) was performed to test the expression of circFndc3b on human retinal vascular endothelial cells (HRVECs) and its exosomes under normal glucose (NG, 5 mmol/L) and high glucose (HG, 30 mmol/L) conditions. MiR39-3p and GAPDH were used as the external and the internal reference, respectively. The sequences of the primers used are listed in Table 1.

**TABLE 1:** Primers used for quantitative PCR (qPCR) validation of the selected circular RNAs (circRNAs).

circBase ID	Primer	Sequence (5′–3′)
hsa_circ_0001535	Forward	TGAGAATGAAGAAAATACCCAGCAC
	Reverse	GATCTATGCTGCTCTGAAGATCAAA
hsa_circ_0001772	Forward	CCAGTACTATCTTCAATCACCTTGC
	Reverse	CATCTGACAAATCCGACTGATTC
hsa_circ_0006156	Forward	AGGGCCATAGTGGTGGAAGTG
	Reverse	CCAGTACTATCTTCAATCACCTTGC

### Uptake of Exosome Assay

Exosomes were labeled with PKH26, according to the manufacturer’s protocol. Briefly, 1 ml Diluent C was mixed with 4 μl PKH26. The exosome suspension was mixed well with the stain solution and then incubated for 10 min. Labeled exosomes were ultracentrifuged at 100,000 × *g* for 70 min and washed with PBS. Then, the purified and labeled exosomes were co-cultured with cells in a humidified incubator at 37°C in 5% CO_2_ for 8 h. The nuclei were dyed with DAPI. The photographs were taken with a confocal laser scanning microscope (CLSM).

### Wound Healing Assay

HRVECs (2.0 × 10^5^) were seeded in a six-well plate cultured with serum-free endothelial cell medium (ECM). A scratch was made with a 200-μl sterile pipette. Subsequently, the cells were washed with PBS and cultured in a humidified incubator at 37°C in 5% CO_2_ for 48 h. The photographs were taken at four time points (0, 12, 24, and 48 h) with a microscope (Nikon TS100, Tokyo, Japan).

### Tube Formation Assay

HRVECs (2.0 × 10^4^) were seeded in 80 μl Matrigel (Matrigel^®^ Matrix) in a 96-well plate. After incubation at 37°C in an atmosphere of 5% CO_2_ for 8 h, the gels were observed with a microscope (at × 4; Nikon TS100). The branch nodes and total tube length were counted using ImageJ software.

### Statistical Analysis

Data were analyzed using SPSS version 19.0. Differences between groups were compared using Student’s *t*-test. A *P*-value < 0.05 was considered statistically significant.

## Results

### Ultrastructure and Characterization of Exosomes

**FIGURE 1:**
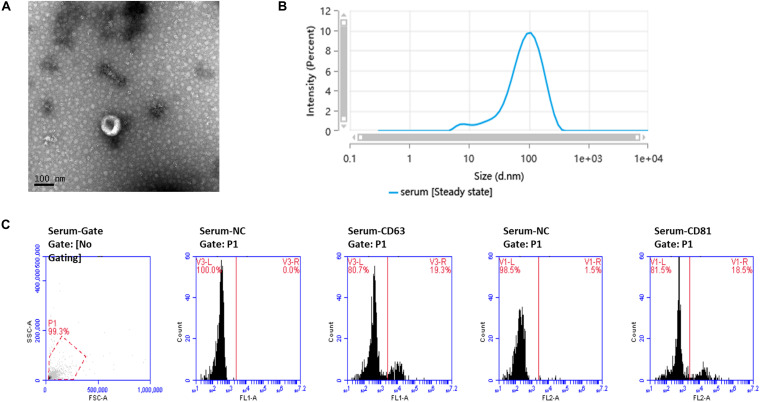
Ultrastructure and characterization of serum-derived exosomes. **(A)** TEM image shows that the exosomes have a cup-shaped structure. *Bar*, 100 nm. **(B)** Of the particles, 89.026% were 20–200 nm in size, as detected by nanoparticle tracking analysis (NTA). **(C)** Flow cytometry analysis of the exosome markers CD63 and CD81, with 19.3 and 18.5% positive rates, respectively.

TEM showed the cup-shaped appearance of the exosomes in this study (Figure 1A). Particle size analysis confirmed that the main peak of particle size, which covered 72.9% of the isolated exosomes in the tested sample, was among the typical size arrangement of exosomes (Figure 1B). FCM analysis showed that serum-derived exosomes contained CD63 and CD81 markers on their surface (Figure 1C).

### DEGs in Serum Exosomes Between the Two Groups

In total, 26 exosomal circRNAs, 106 miRNAs, and 2,264 mRNAs with DEGs were identified in the PDR group compared with the age-matched senile cataract group (fold change > 1, *P* < 0.05). The scatter plot and hierarchical clustering plot visualized the variations in the expression profiles of the circRNAs (Supplementary Figure 1), miRNAs (Supplementary Figure 2), and mRNAs (Supplementary Figure 3) between the two groups.

### Construction of the ceRNA Network

**FIGURE 2:**
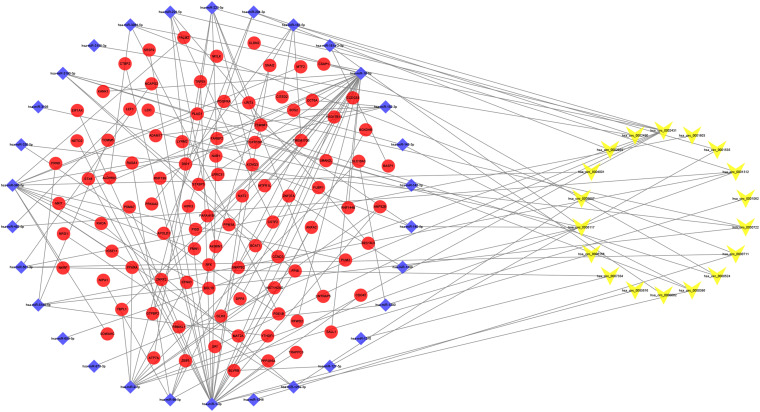
Circular RNA (circRNA)–microRNA (miRNA)–messenger RNA (mRNA) competitive endogenous RNA (ceRNA) network. *Yellow-V*, *blue diamonds*, and *red ellipses* represent circRNAs, miRNAs, and mRNAs, respectively.

We identified 865 circRNA–miRNA pairs, including 18 DEcircRNAs and 744 miRNAs. After intersecting with the 106 DEmiRNAs, only 30 miRNAs remained. Subsequently, the target genes were predicted for these 30 miRNAs by the TargetScan, miRDB, and miRTarBase databases, which were then overlapped with the 2,264 DEmRNAs collected from the RNA-seq, resulting in 98 genes shared. Based on the combination of the above data, a ceRNA network related to PDR was established (Figure 2).

### PPI Network Construction and Functional Enrichment Analysis

**FIGURE 3:**
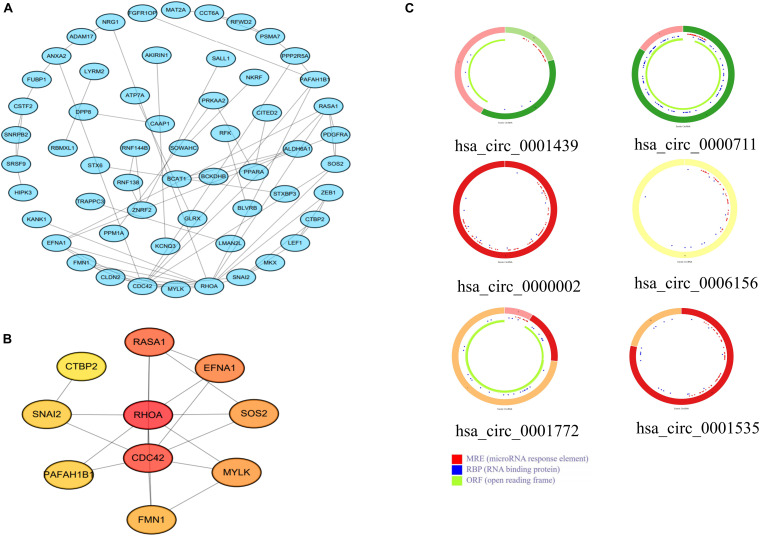
**(A)** A protein–protein interaction (PPI) network was established using the 98 common differentially expressed messenger RNAs (DEmRNAs). **(B)** PPI network of the 10 hub genes extracted from **(A)**. **(C)** Structural patterns of the top six dysregulated circular RNAs (circRNAs) **(C)**.

**FIGURE 4:**
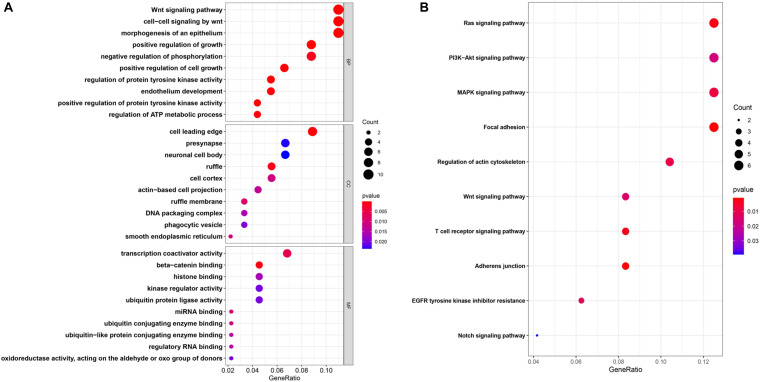
**(A,B)** Enrichment of the top 10 Gene Ontology (GO) terms **(A)** and Kyoto Encyclopedia of Genes and Genomes (KEGG) pathways **(B)** of the differentially expressed mRNAs. The *color of the dots* changes gradually from *blue* to *red* in ascending order according to the adjusted *P*-values. The *size of the node* represents the number of counts.

To further explore the interactions among the 98 common DEGs, we established the PPI network (Figure 3A). The PPI network contained 98 nodes and 68 edges; the node degree and the local clustering coefficient were 1.39 and 0.413, respectively. According to the Matthews correlation coefficient (MCC) score, 10 hub genes were identified using the Cytoscape software. These screened hub genes were *RhoA*, *Cdc42*, and *RASA1*, among others (Figure 3B). The structural patterns and basic information on the characteristics of the top six dysregulated exosomal circRNAs are summarized in Figure 3C and Table 2. GO enrichment, including biological process (BP), molecular function (MF), and cell component (CC), was performed to determine the biological role of the DEmRNAs. As shown in the bubble diagram, the most significant terms in BP, CC, and MF were “Wnt signaling pathway,” “cell leading edge,” and “transcription coactivator activity,” respectively (Figure 4A). Moreover, we analyzed the KEGG pathways of the DEGs. The results showed the top 10 KEGG pathways (Figure 4B) mainly enriched in the RAS signaling pathway, PI3K–AKT signaling pathway, mitogen-activated kinase (MAPK) signaling pathway, and focal adhesion.

**TABLE 2:** Basic characteristics of the top six dysregulated exosomal circular RNAs (circRNAs).

circRNA ID	Gene name	Strand	Genomic length	Spliced sequence length
hsa_circ_0001535	*FAM13B*	–	3,059	331
hsa_circ_0001772	*RBM33*	+	8,042	445
hsa_circ_0006156	*FNDC3B*	+	4,009	526
hsa_circ_0001439	*SCLT1*	–	11,710	396
hsa_circ_0000002	*SDF4*	–	725	251
hsa_circ_0000711	*NFATC3*	+	4,624	1,298

### Validation of Exosomal circFndc3b Functions

**FIGURE 5:**
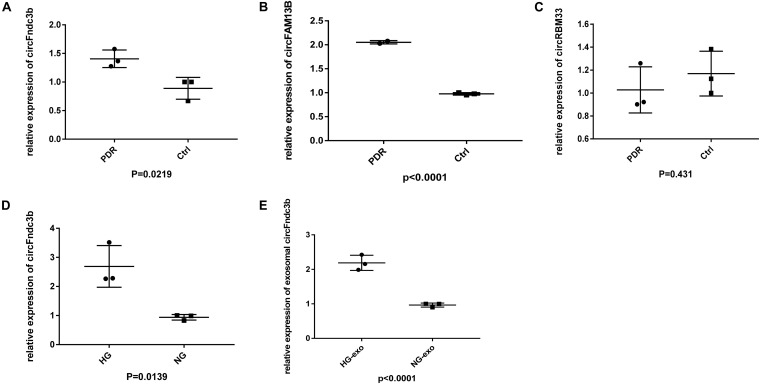
Validation of exosomal circular RNAs (circRNAs). **(A–C)** Quantitative real-time PCR (qRT-PCR) verified the expressions of exosomal circFndc3b **(A)**, circFAM13B **(B)**, and circRBM33 **(C)** in proliferative diabetic retinopathy (PDR) and control serum exosomes. **(D,E)** Expression of circFndc3b on the high-glucose (HG) and negative control (NG) conditions at the cellular **(D)** and exosomal **(E)** levels in human retinal vascular endothelial cells (HRVECs).

We collected another six samples to verify the expressions of the most upregulated circRNAs based on RNA-seq. Among the three selected circRNAs, the expressions of circFndc3b (Figure 5A) and circFAM13B (Figure 5B) were in line with the RNA-seq results, and there was no significant difference in circRBM33 between the PDR and age-matched senile cataract groups (Figure 5C). qRT-PCR indicated that the expressions of HRVECs–circFndc3b on HG stress were significantly higher than those in NG at the cellular (Figure 5D) and exosomal levels (Figure 5E).

**FIGURE 6:**
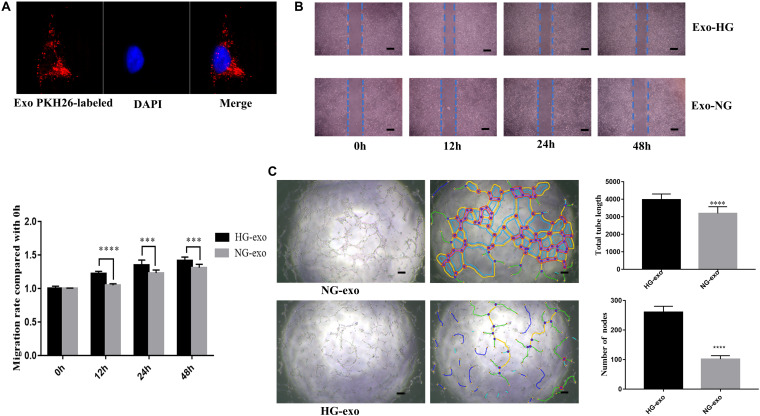
Function of exosomes derived from high-glucose (HG)-induced human retinal vascular endothelial cells (HRVECs) on angiogenesis *in vitro*. **(A)** Endothelial cells phagocytosed exosomes labeled by PKH26. **(B,C)** Exosomes derived from HG-induced HRVECs can enhance the migration **(B)** and tube formation ability **(C)** of endothelial cells (ECs) in comparison to negative control (NG) exosomes. Representative images of wound healing and tube formation are shown along with quantitative data (*n* = 3). *Scale bar*, 100 μm. ***P < 0.0002, ****P < 0.0001.

**FIGURE 7:**
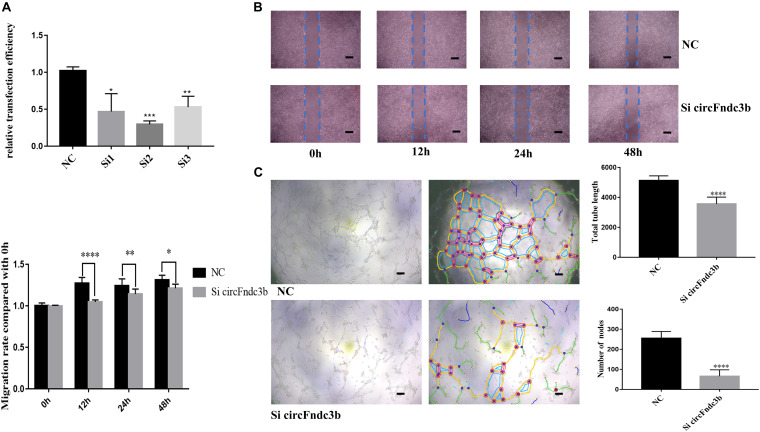
Function of circFndc3b in angiogenesis *in vitro*. **(A)** Small interfering RNA 2 had the greatest interference efficiency. **(B,C)** circFndc3b knockdown can reduce the migration **(B)** and tube formation ability **(C)** of endothelial cells (ECs) in comparison to the negative control. Representative images of wound healing and tube formation are shown along with quantitative data (*n* = 3). *Scale bar*, 100 μm. *P < 0.0332, **P < 0.0021, ***P < 0.0002, ****P < 0.0001.

Next, the purified exosomes marked with PKH26 were uptaken by HRVECs (Figure 6A). The wound healing assay indicated that the exosomes derived from HG-induced HRVECs can increase the migration ability of endothelial cells (ECs) in comparison to NG exosomes (Figure 6B). Results of the tube formation assay suggested that the number of nodes and total tube length were significantly higher in the HG-exo group than those in the NG-exo group (Figure 6C). The above findings reflected that circFndc3b was significantly overexpressed under the HG condition at the exosomal and cellular levels. Meanwhile, HG exosomes have a potential function to promote angiogenesis *in vitro*. Thus, we determined to explore whether circFndc3b could modulate the angiogenic ability of HRVECs. Three small interfering RNAs (siRNAs) were synthesized, and siRNA2 had the greatest interference efficiency when compared with the negative control (NC) by qRT-PCR (Figure 7A). circFndc3b knockdown could decrease the migration of HRVECs, as detected by the wound healing assay (Figure 7B). The tube formation assay showed that the number of nodes and total tube length were significantly lower in the knockdown group than those in the NC group (Figure 7C). The results described collectively suggest that exosomal circFndc3b plays a crucial rule in angiogenesis *in vitro*.

## Discussion

In our study, differentially expressed exosomal circRNAs were identified *via* high-throughput sequencing from 10 PDR patients and 10 age-matched patients with senile cataract and were validated by PCR. Bioinformatics analysis indicated that 18 DEcircRNAs and 30 miRNAs may be closely related to the pathogenesis of PDR. Furthermore, circFndc3b and exosomes derived from HG-induced HRVECs were identified as having the capability to facilitate angiogenesis. Based on the joint analysis of multiple databases, the PPI network identified 10 hub genes, such as *RhoA*, *Cdc42*, and *RASA1*, involved in the regulation of the PDR process. Of note is that previous research also revealed these hub genes to be responsible for the angiogenesis of PDR. For example, Lu et al. (2015) systematically described that RhoA/mDia-1/profilin-1 signaling targets microvascular endothelial dysfunction in DR. Cell division cycle 42 (Cdc42), a small GTPase of the Rho family, can regulate insulin secretion and diabetes-associated diseases such as diabetic nephropathy (DN) and diabetic foot (Huang et al., 2019). KEGG and GO analyses are also important in bioinformatics analysis, providing new perspectives on the comprehensive function of DEGs. The RAS, PI3K–AKT, and MAPK signaling pathways were the top three enriched signaling pathways in KEGG. Interestingly, early researches have shown that these pathways are also involved in maintaining angiogenic homeostasis (Kowluru and Kowluru, 2007; Jin et al., 2013). Westenskow et al. (2013) elaborated that RAS signaling pathway inhibition prevents angiogenesis by repressing endothelial cell sprouting.

Exosomes derived from peripheral blood have recently emerged as an important class of circulating biomarkers for diseases (Jia et al., 2017; Mirzakhani et al., 2019). Exosomes contain a multitude of biological molecules (proteins, nucleic acids, lipids, etc.) from parental cells, with high stability and specificity. Thus, exosomes have greater advantages as progressive and prognostic biomarkers than do traditional test methods such as circulating tumor cells (CTCs) and circulating tumor DNA (ctDNA) (Heitzer et al., 2019). Nowadays, a host of serum exosomal circRNAs were considered as promising markers and intervention targets (Pan et al., 2019; Xian et al., 2020). For instance, exosomal circ-IARS was higher than those in the control groups both in pancreatic cancer tissues and in plasma exosomes, indicating that exosomal circRNA could be a meaningful marker for pancreatic ductal adenocarcinoma (PDAC) (Li et al., 2018). Besides, Akbar et al. (2019) critically illustrated the potential application of cargoes carried by exosomes in metabolic diseases, including DR. Previously, studies have demonstrated that several circRNAs, such as cZNF609 and circZNF532, also play important roles in the regulation of diabetes-induced retinal pericyte degeneration and vascular dysfunction (Liu et al., 2017; Jiang et al., 2020).

In this study, circFndc3b and circFAM13B are two novel circRNAs that were first discovered in PDR. We found that circFndc3b and exosomes derived from HG-induced endothelial cells have the capability to facilitate angiogenesis *in vitro*. In the cardiovascular system, circFndc3b can reduce cardiomyocyte apoptosis and fibrosis and enhance angiogenesis by promoting VEGF expression and signaling *via* its interaction with the RNA-binding protein fused in sarcoma (FUS) after myocardial infarction (MI) (Garikipati et al., 2019). In the retina, however, the retinal angiogenesis facilitated by exosomal circFndc3b is abnormal, with a limited capacity to perform normal physical functions and higher possibility of leakage and hemorrhage.

There are several limitations in our study. Firstly, we showed here the preliminary findings on exosomal circRNAs abnormally regulated in PDR serum. Further support is needed through *in vivo* experiments and clinical data, especially concerning circFndc3b. Secondly, DR is a complex pathological process that can be provoked by multiple pathological factors, such as inflammation, oxidative stress, and apoptosis, which constitute a complicated crossover network and affect each other to continuously worsen microvascular damage. HG stress is the initiator of DR. We only employed HG stress to simulate the DR environment, which is not appropriate. In addition, the specific underling mechanisms of the upstream and downstream pathways of circFndc3b need to be studied in detail. Finally, larger clinical samples and epidemiological investigations are needed to determine the underlying roles of aberrant exosomal circRNAs as progression or prognostic biomarkers for PDR.

## Conclusion

In summary, the aberrant profiles of exosomal circRNAs from PDR serum and their bioinformatics analysis will assist in understanding the underlying molecular mechanisms of the circRNAs involved in the development and progression of PDR. Future exploration of the mysterious roles of exosomes and exosomal circRNAs may shed light for thorough understanding of the biological functions of exosomal circRNAs in PDR.

## Data Availability Statement

The datasets presented in this study can be found in online repositories. The names of the repository/repositories and accession number(s) can be found below: NCBI GEO, accession no: GSE178721.

## Ethics Statement

The studies involving human participants were reviewed and approved by the Research and Ethical Committee of the First Affiliated Hospital of Nanjing Medical University. The patients/participants provided their written informed consent to participate in this study.

## Author Contributions

XL and ZH designed the research project. PX organized the project. XL, JW, HQ, ZZ, and YW designed the experiments and statistical analysis. XL wrote the first draft of the manuscript. ZH and PX reviewed and critiqued the manuscript. All authors contributed to the article and approved the submitted version.

## Conflict of Interest

The authors declare that the research was conducted in the absence of any commercial or financial relationships that could be construed as a potential conflict of interest.

## Publisher’s Note

All claims expressed in this article are solely those of the authors and do not necessarily represent those of their affiliated organizations, or those of the publisher, the editors and the reviewers. Any product that may be evaluated in this article, or claim that may be made by its manufacturer, is not guaranteed or endorsed by the publisher.

## Footnotes

1
http://gb.whu.edu.cn/CSCD/

2
www.cytoscape.org/

3
http://string-db.org/

## Supplementary Material

Supplementary Figure 1 The scatter plot and hierarchical clustering analysis showed the expression of circRNAs between the two groups in serum exosomes.

Supplementary Figure 2 The scatter plot and hierarchical clustering analysis showed the expression of miRNAs between the two groups in serum exosomes.

Supplementary Figure 3 The scatter plot and hierarchical clustering analysis showed the expression of mRNAs between the two groups in serum exosomes.
