# Corrigendum: Serum exosomal circular RNA expression profile and regulative role in proliferative diabetic retinopathy

**DOI:** 10.3389/fgene.2024.1497882

**Published:** 2024-11-19

**Authors:** Xinsheng Li, Jingfan Wang, Huiming Qian, Yan Wu, Zhengyu Zhang, Zizhong Hu, Ping Xie

**Affiliations:** Department of Ophthalmology, The First Affiliated Hospital of Nanjing Medical University, Nanjing, China

**Keywords:** proliferative diabetic retinopathy, exosome, circular RNA, angiogenesis, bioinformatics analysis

In the published article, there was an error in “Figure 7 Function of circFndc3b in angiogenesis *in vitro*” as published. This occurred during article production, when the two panels on the top left were mistakenly duplicated those from the equivalent positions in **Figure 6B**.

**FIGURE 7:**
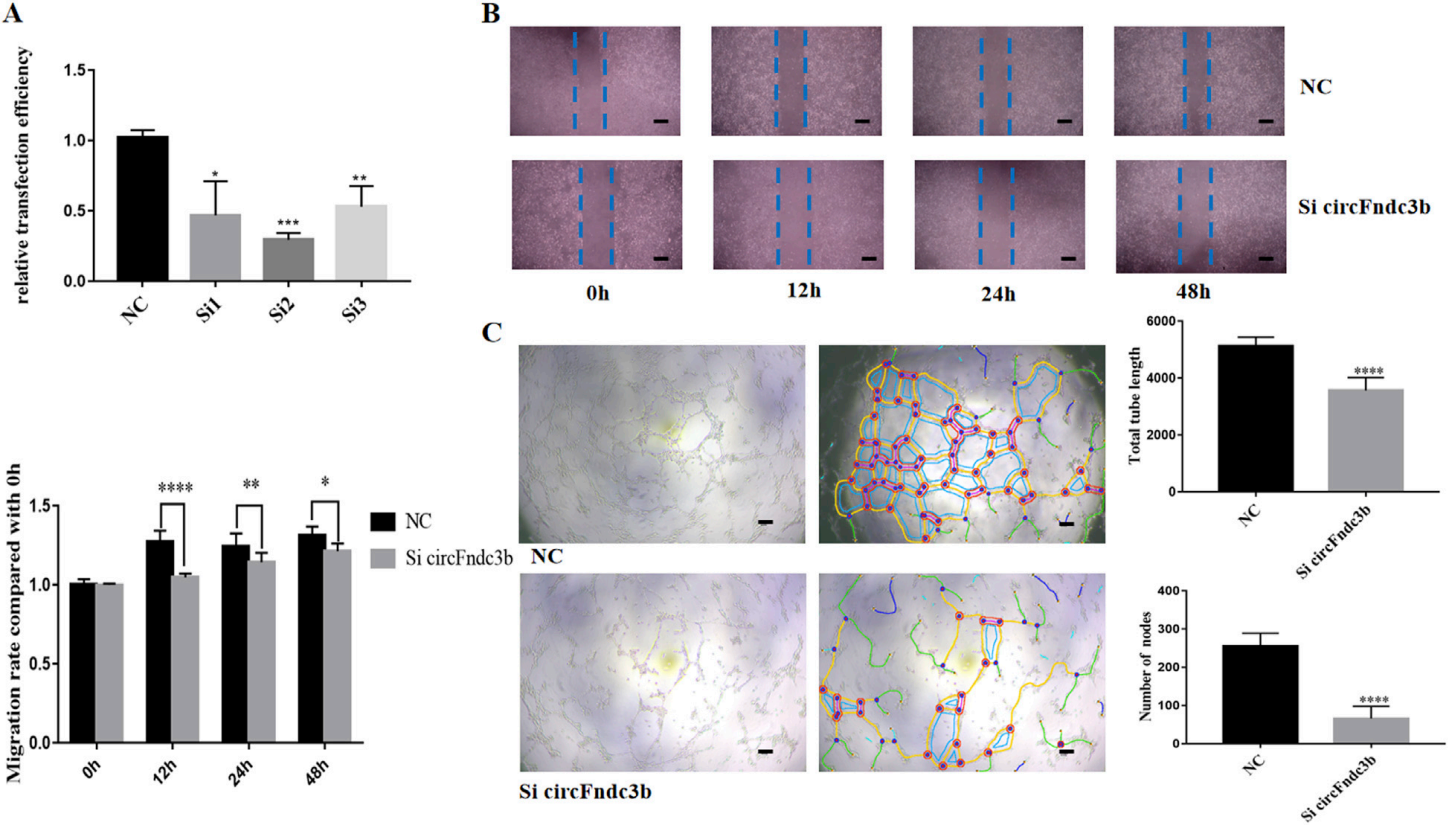
Function of circFndc3b in angiogenesis *in vitro*: **(A)** Small interfering RNA 2 had the greatest interference efficiency. **(B, C)** CircFndc3b knockdown can reduce the migration **(B)** and tube formation ability **(C)** of endothelial cells (ECs) in comparison to the negative control. Representative images of wound healing and tube formation are shown along with quantitative data (n = 3). Scale bar, 100 µm. *P < 0.0332, **P < 0.0021, ***P < 0.0002, ****P < 0.0001.

The revised Figure 7 is presented below.

The authors apologize for this error and state that this does not change the scientific conclusions of the article in any way. The original article has been updated.

